# Supplementary figures and images for: Knockout of cyclin-dependent kinases 8 and 19 leads to depletion of cyclin C and suppresses spermatogenesis and male fertility in mice
Source: eLife. 2025 Apr 2;13:RP96465. doi: 10.7554/eLife.96465 (PMC11964450; doi:10.7554/eLife.96465)

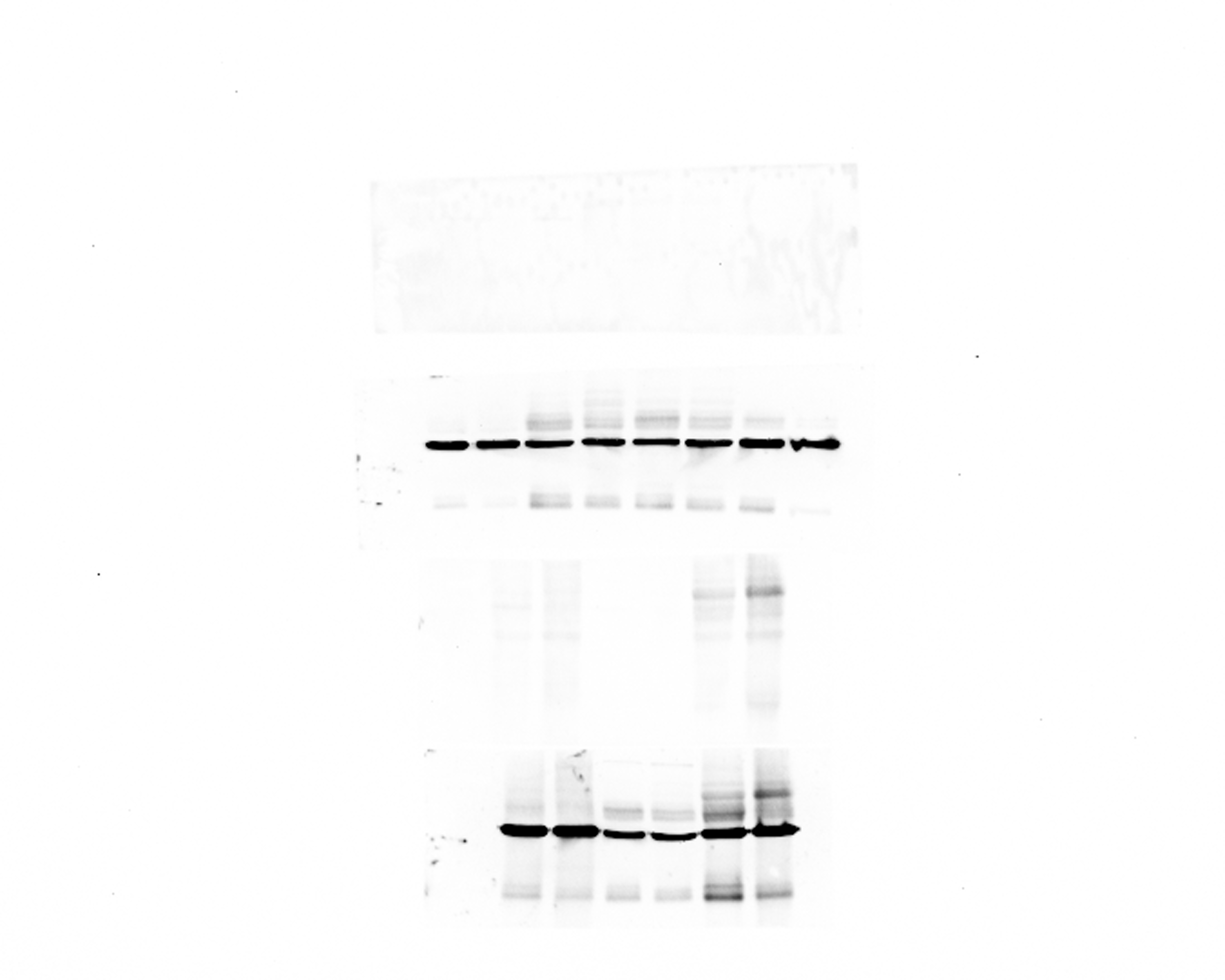

Supplement: Figure 1—source data 2. [file elife-96465-fig1-data2.zip › Figure 1 - source data 2. Original files for western blot analysis displayed in Figure 1B/b-Actin.tif]

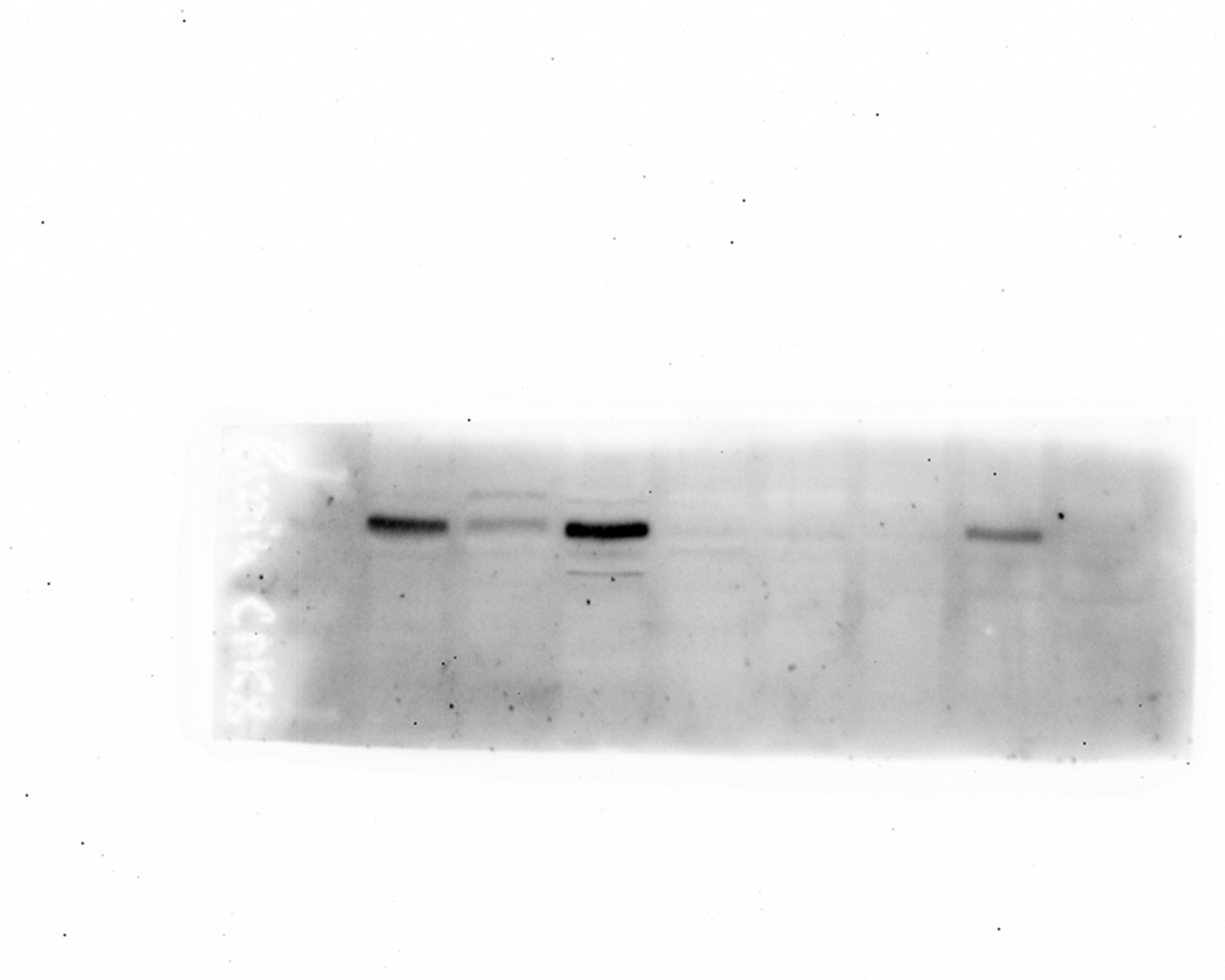

Supplement: Figure 1—source data 2. [file elife-96465-fig1-data2.zip › Figure 1 - source data 2. Original files for western blot analysis displayed in Figure 1B/CDK8.tif]

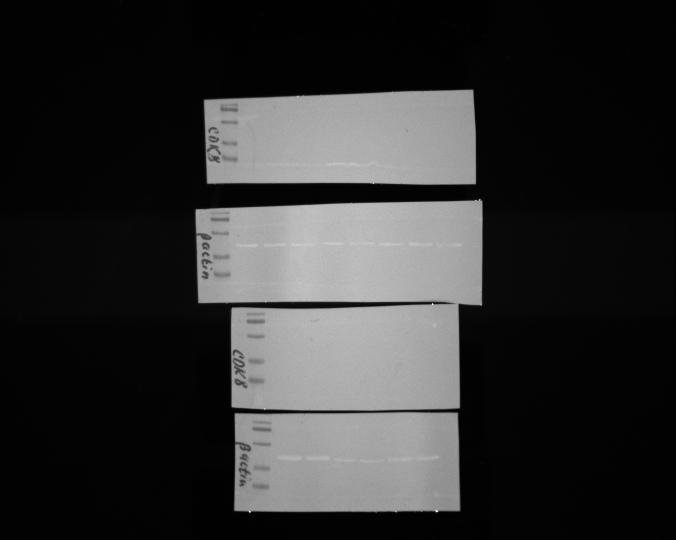

Supplement: Figure 1—source data 2. [file elife-96465-fig1-data2.zip › Figure 1 - source data 2. Original files for western blot analysis displayed in Figure 1B/CDK8_b-actin (Memranes).tif]

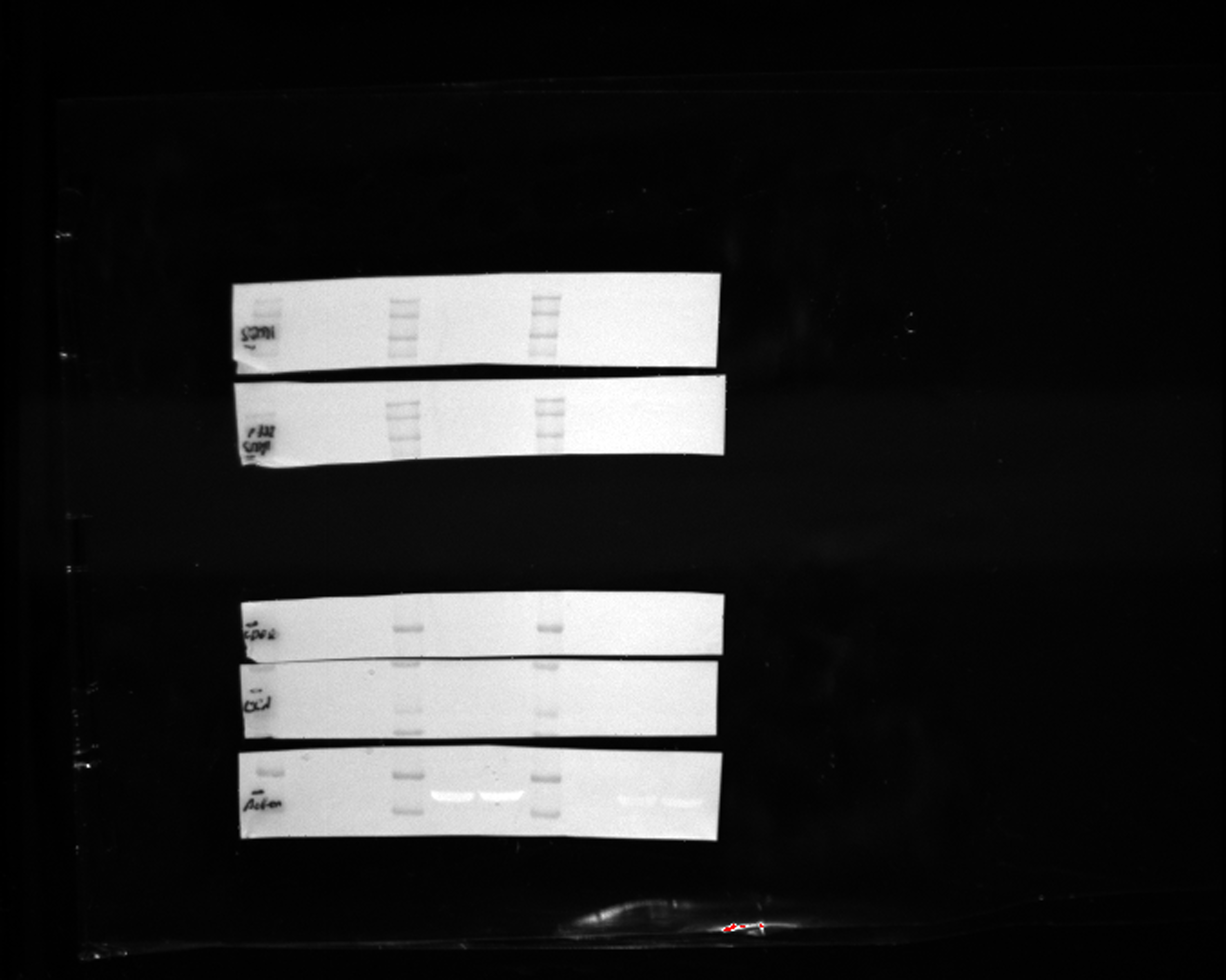

Supplement: Figure 1—figure supplement 1—source data 2. [file elife-96465-fig1-figsupp1-data2.zip › Figure 1-figure supplement 1-source data 2. Original files for gel analysis displayed in Sup.Fig 1B/b-actin (membrane).tif]

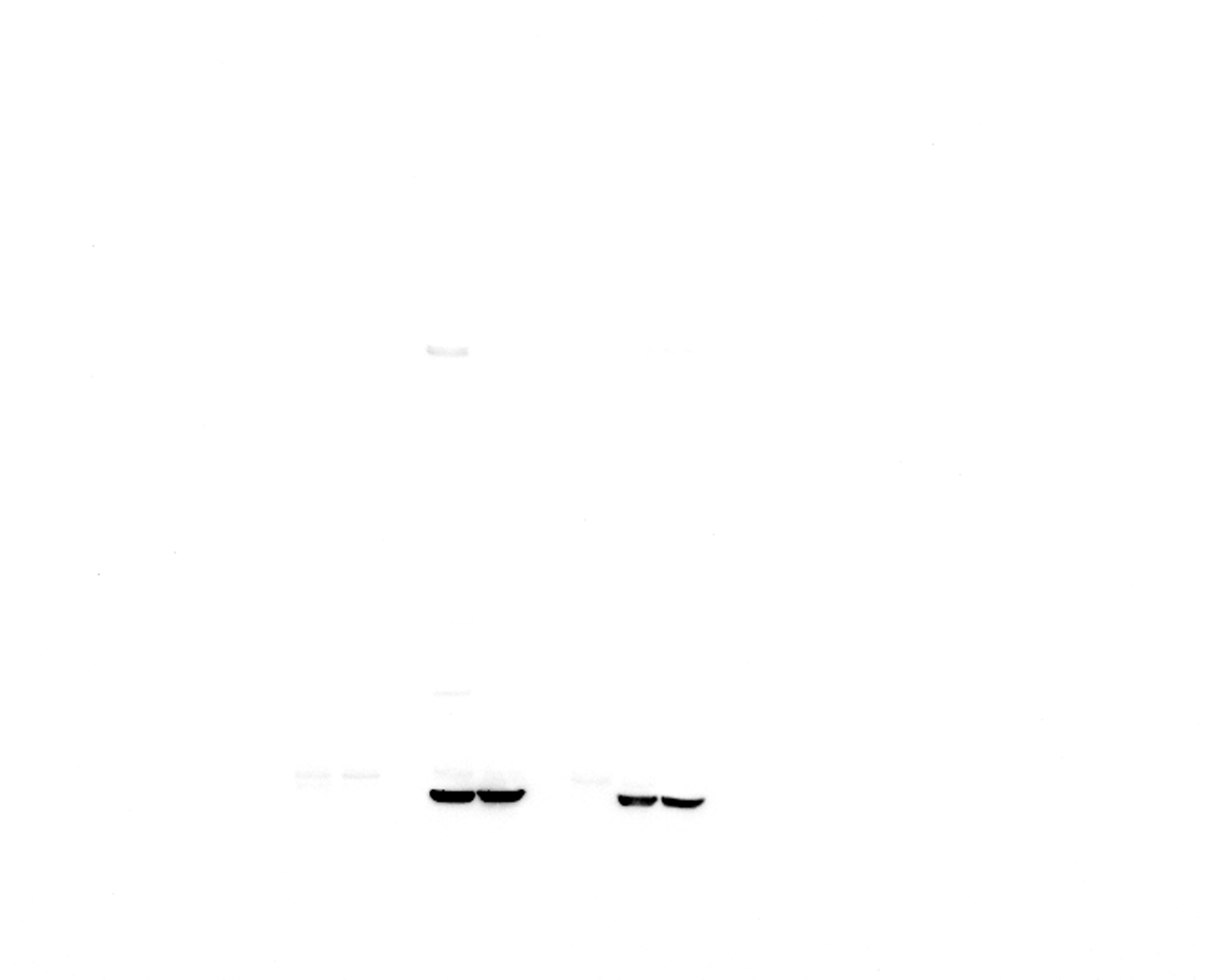

Supplement: Figure 1—figure supplement 1—source data 2. [file elife-96465-fig1-figsupp1-data2.zip › Figure 1-figure supplement 1-source data 2. Original files for gel analysis displayed in Sup.Fig 1B/b-actin.tif]

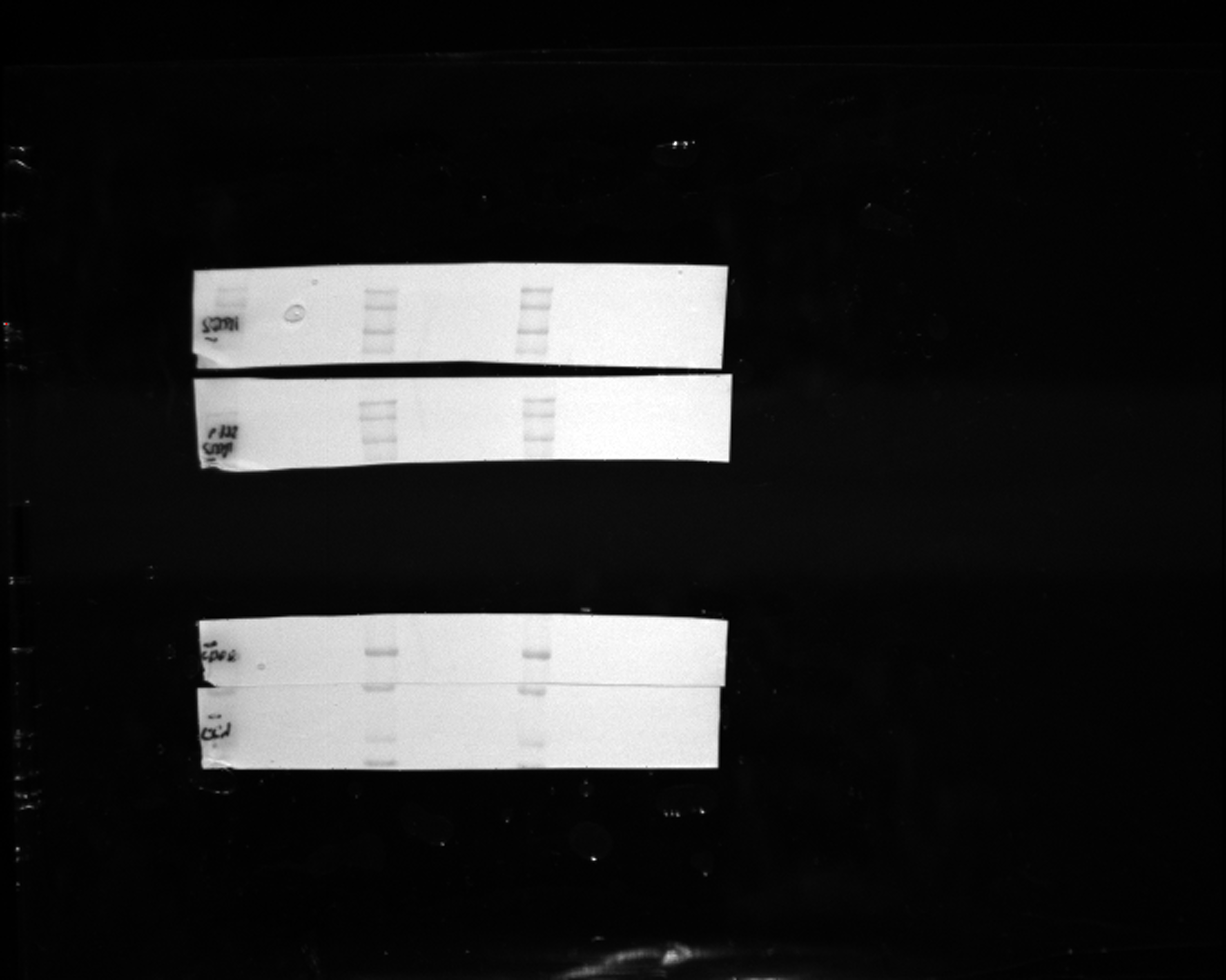

Supplement: Figure 1—figure supplement 1—source data 2. [file elife-96465-fig1-figsupp1-data2.zip › Figure 1-figure supplement 1-source data 2. Original files for gel analysis displayed in Sup.Fig 1B/CDK8 + CCNC (membrane).tif]

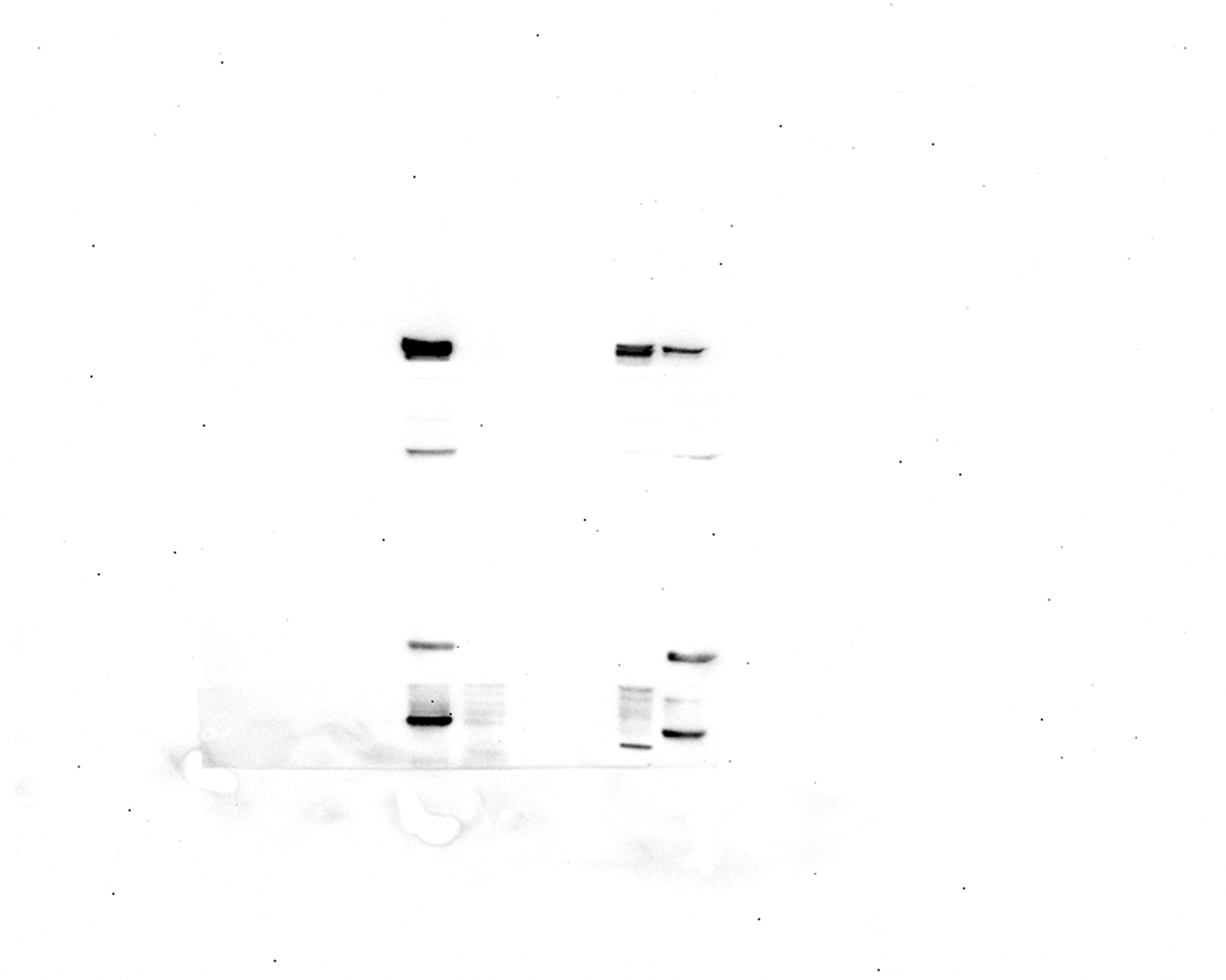

Supplement: Figure 1—figure supplement 1—source data 2. [file elife-96465-fig1-figsupp1-data2.zip › Figure 1-figure supplement 1-source data 2. Original files for gel analysis displayed in Sup.Fig 1B/CDK8 + CCNC.tif]

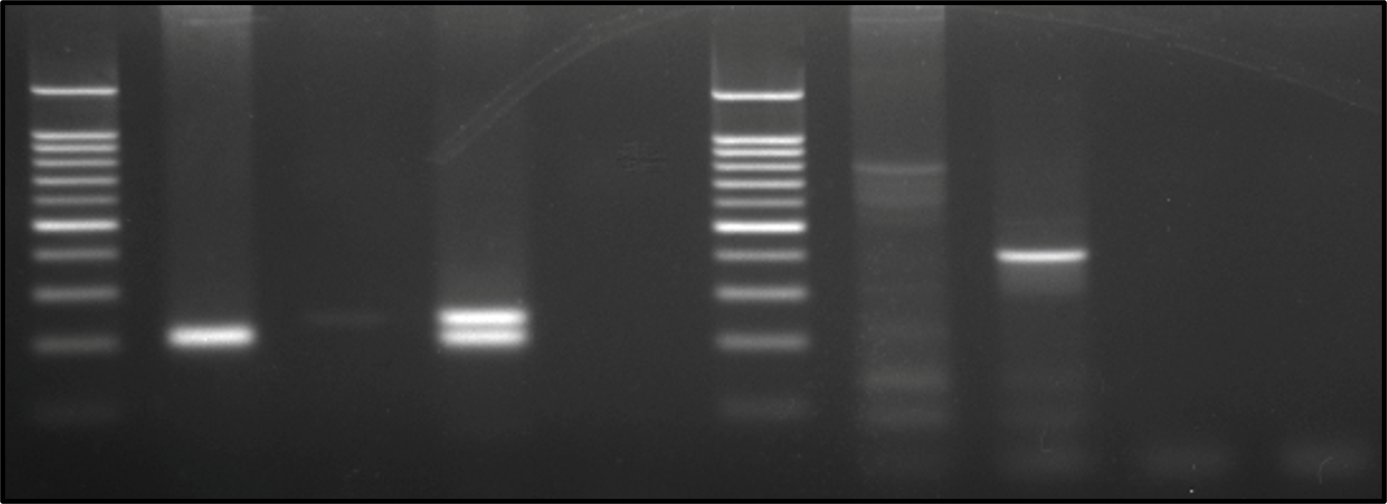

Supplement: Figure 1—figure supplement 1—source data 2. [file elife-96465-fig1-figsupp1-data2.zip › Figure 1-figure supplement 1-source data 2. Original files for gel analysis displayed in Sup.Fig 1B/Fig1_suppl2B_original gel.tif]

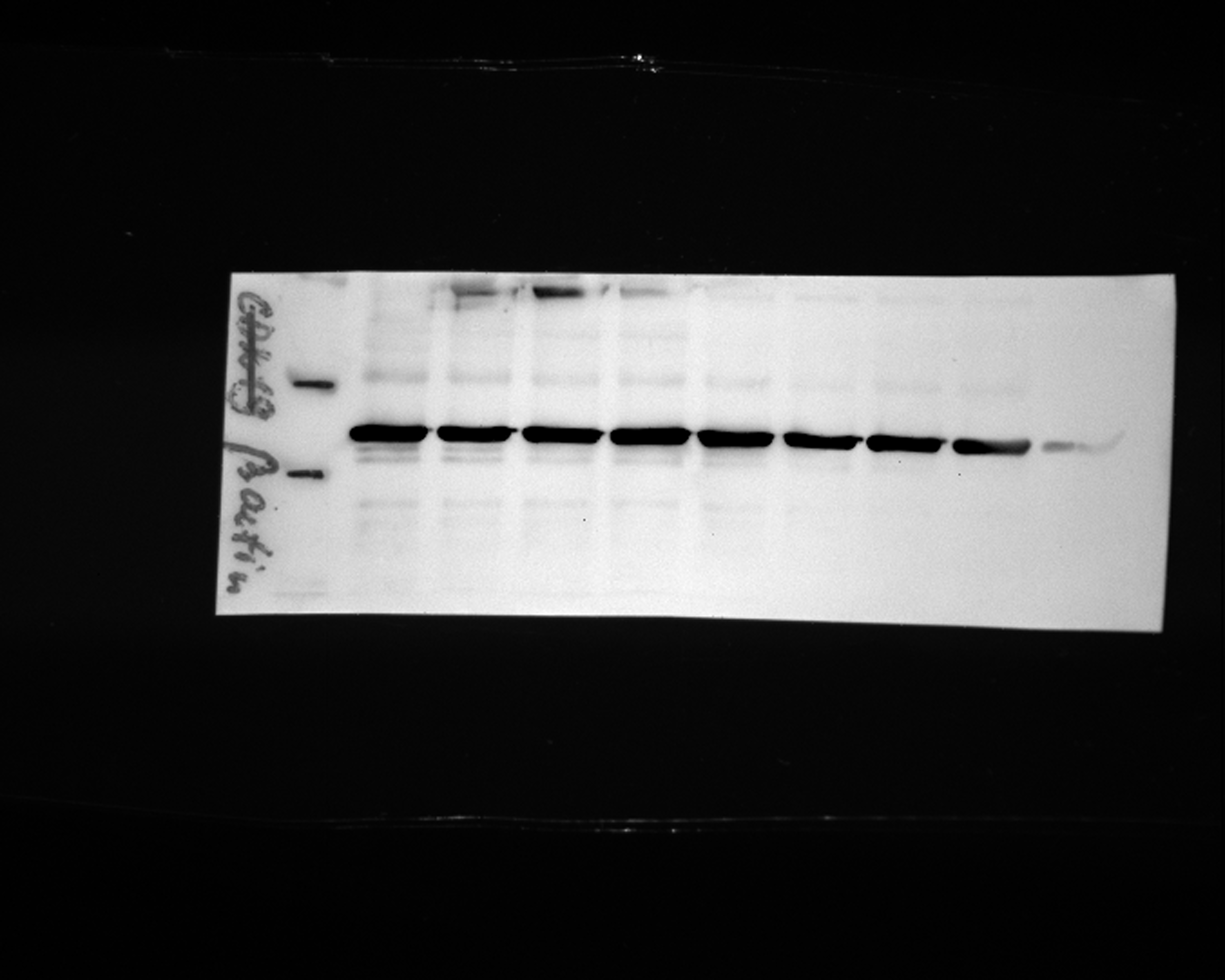

Supplement: Figure 2—source data 2. [file elife-96465-fig2-data2.zip › Figure 2 - source data 2. Original files for western blot analysis displayed in Figure 2D/b-actin.tif]

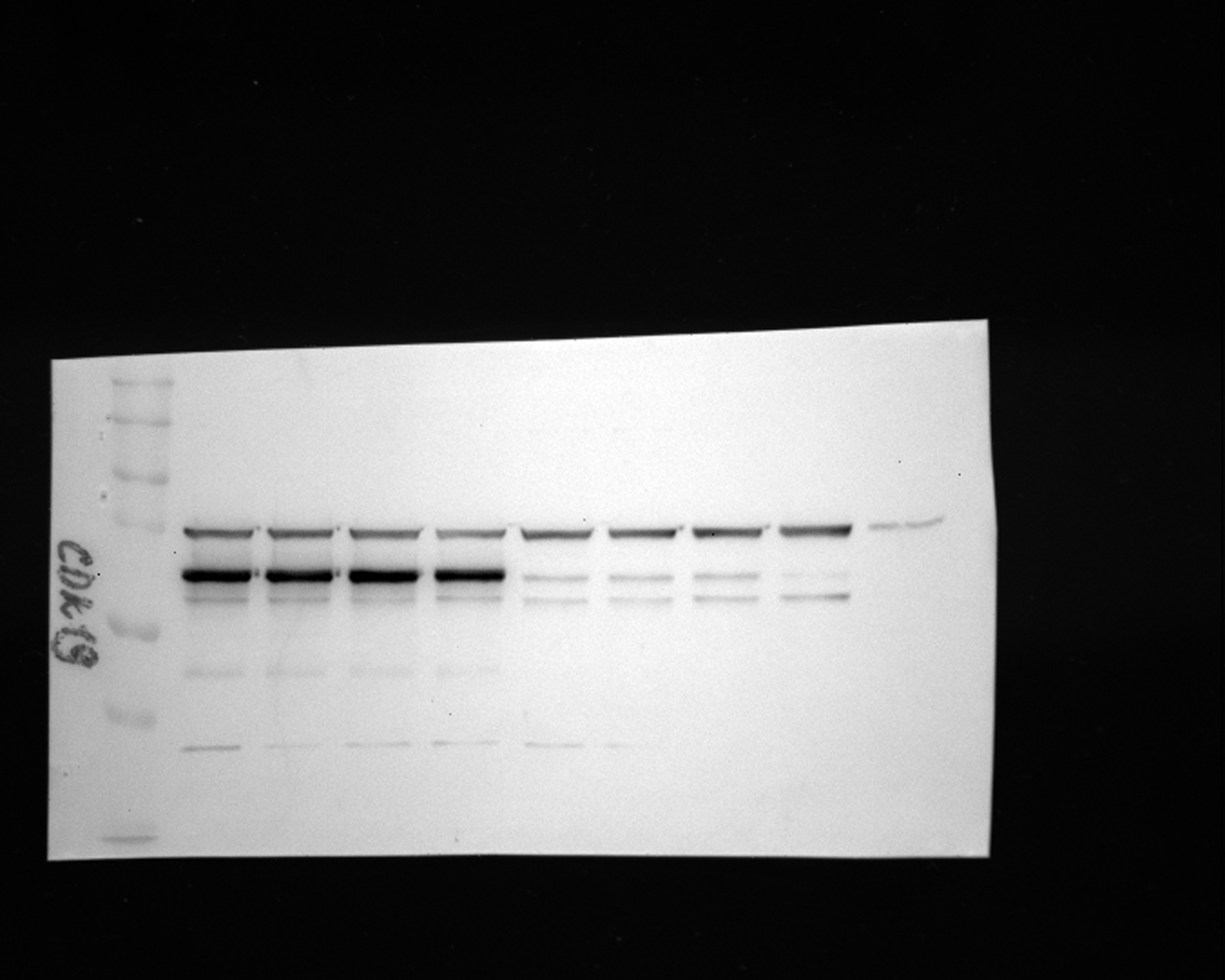

Supplement: Figure 2—source data 2. [file elife-96465-fig2-data2.zip › Figure 2 - source data 2. Original files for western blot analysis displayed in Figure 2D/CDK19.tif]

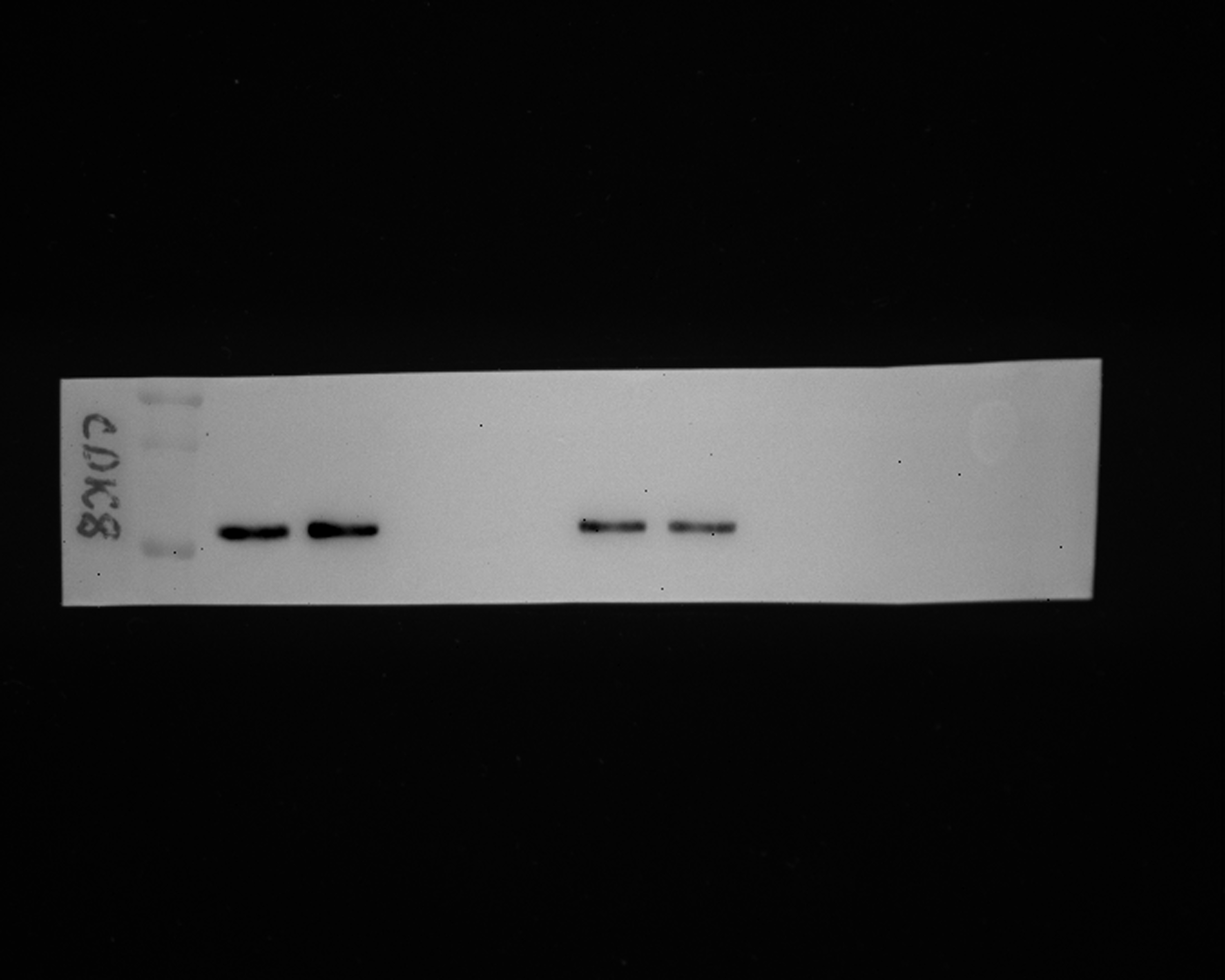

Supplement: Figure 2—source data 2. [file elife-96465-fig2-data2.zip › Figure 2 - source data 2. Original files for western blot analysis displayed in Figure 2D/CDK8.tif]

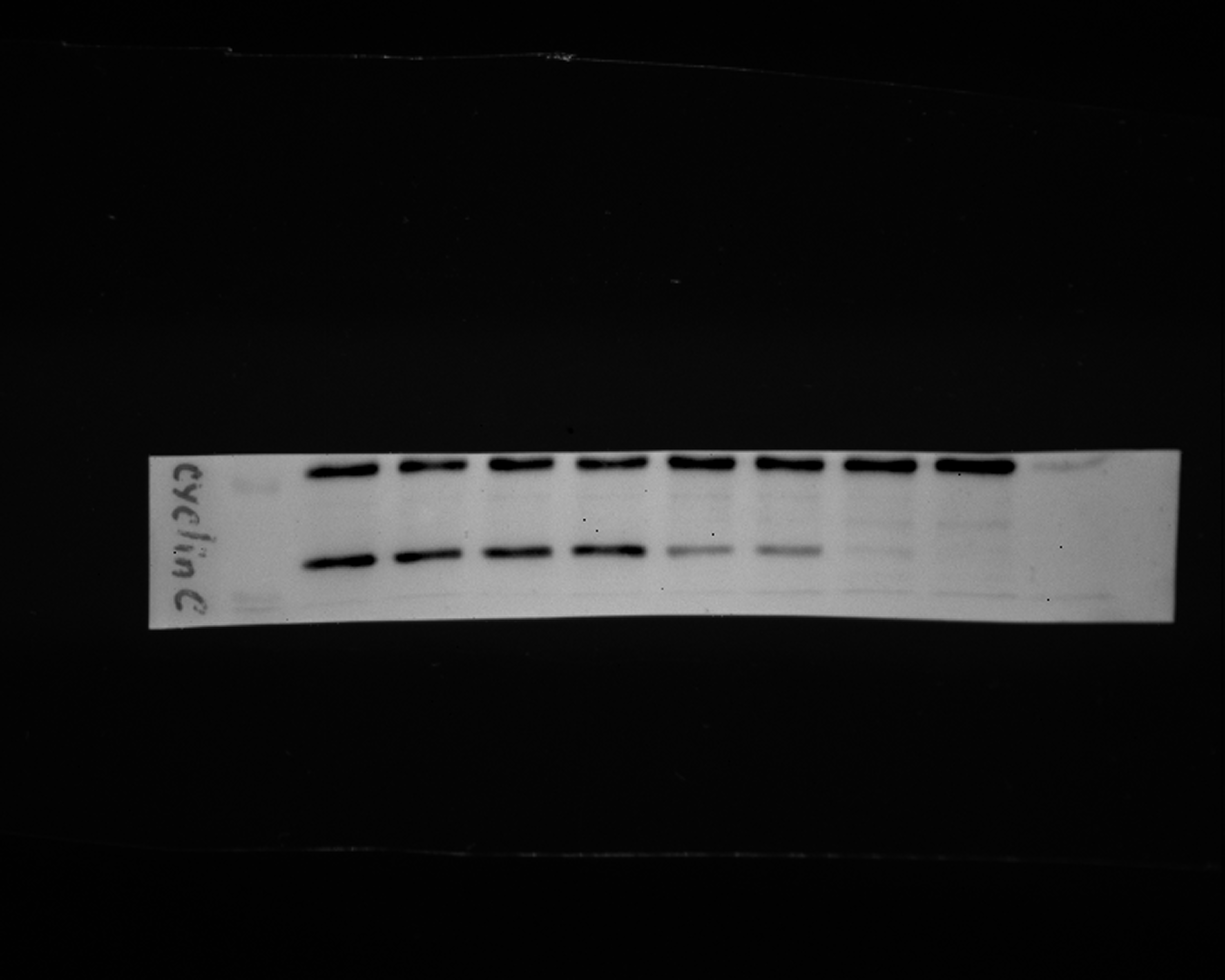

Supplement: Figure 2—source data 2. [file elife-96465-fig2-data2.zip › Figure 2 - source data 2. Original files for western blot analysis displayed in Figure 2D/Cyclin C.tif]

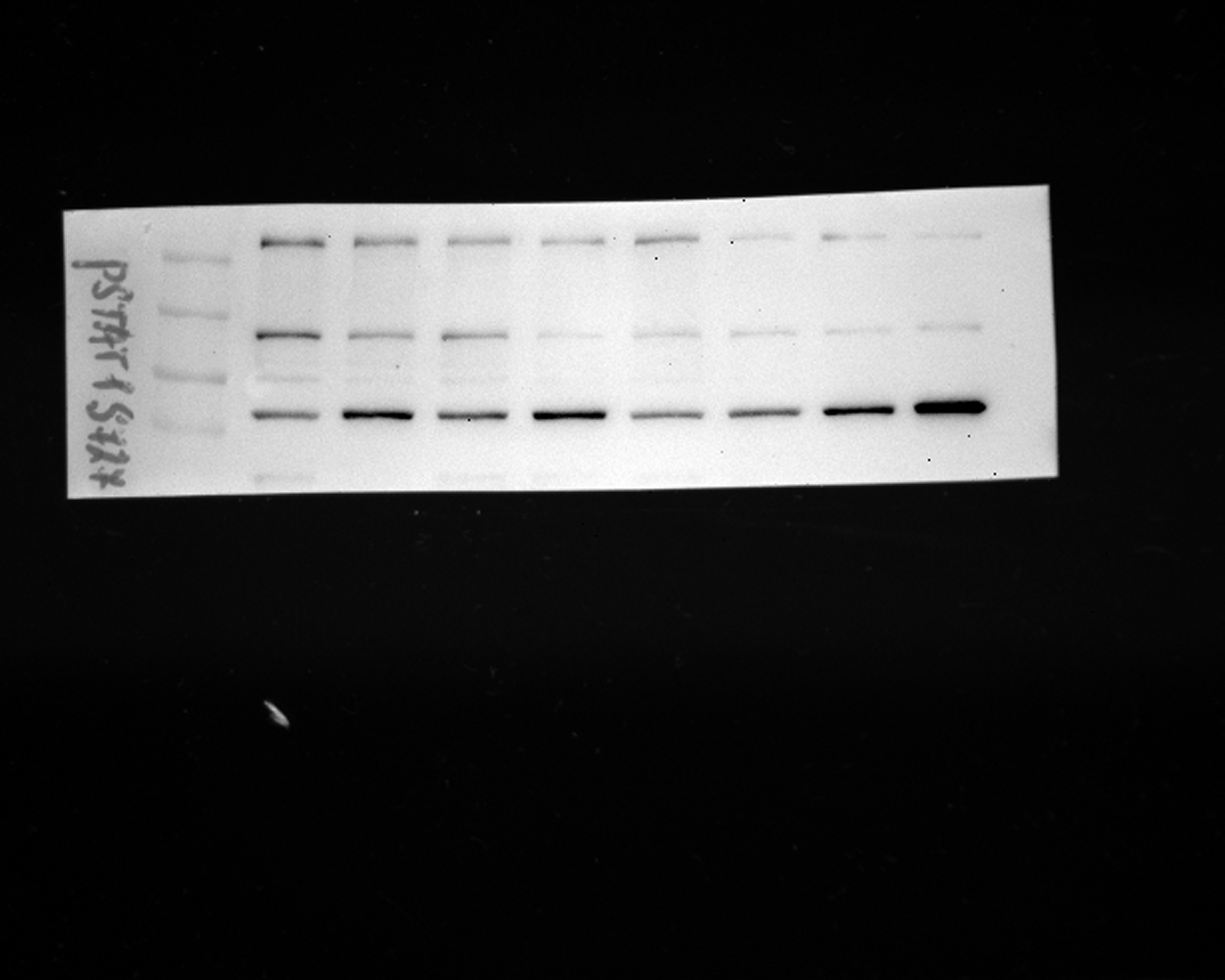

Supplement: Figure 2—source data 2. [file elife-96465-fig2-data2.zip › Figure 2 - source data 2. Original files for western blot analysis displayed in Figure 2D/pSTAT1 S727.tif]

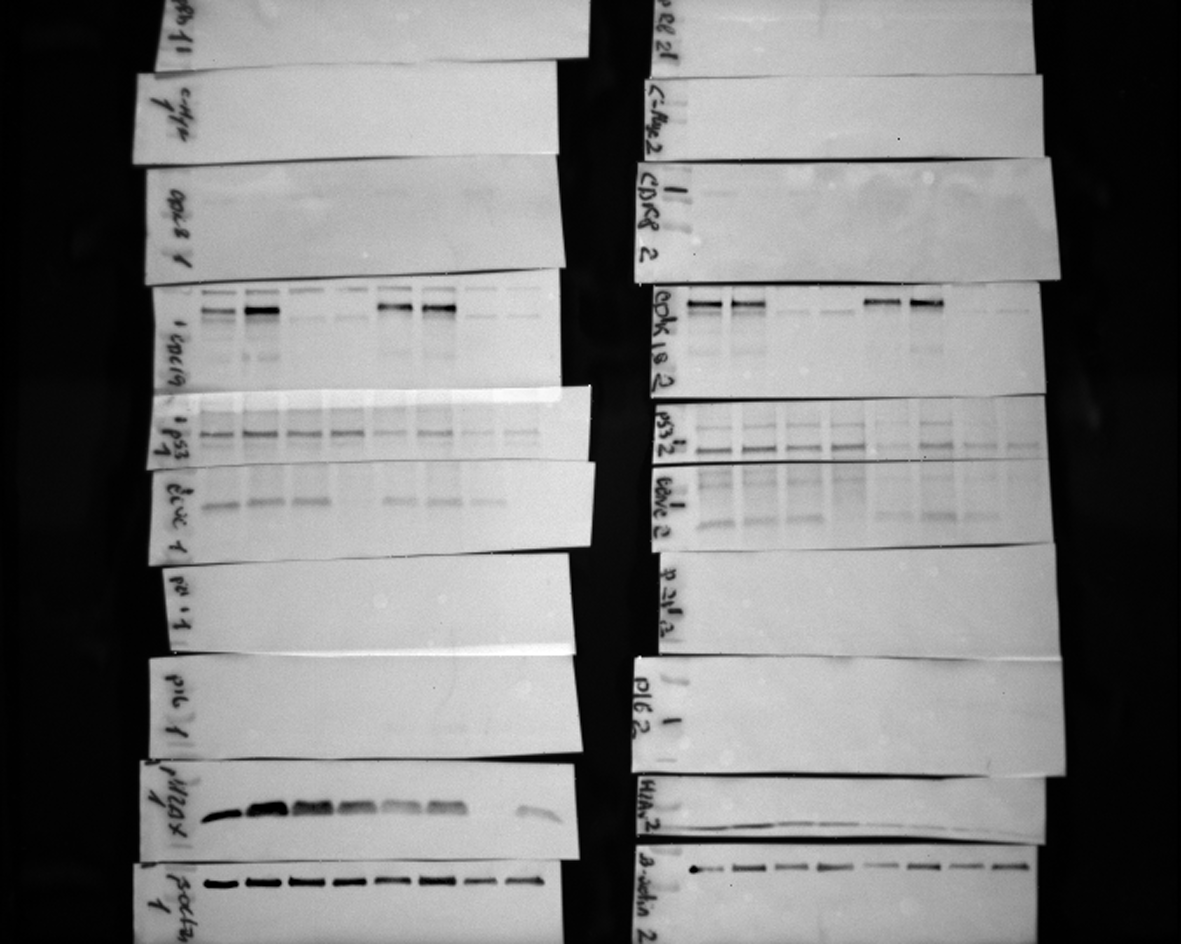

Supplement: Figure 2—figure supplement 1—source data 2. [file elife-96465-fig2-figsupp1-data2.zip › Figure 2-figure supplement 1-source data 2. Original files for western blot analysis displayed in Sup.Fig 2B/CDK19 + b-actin.tif]

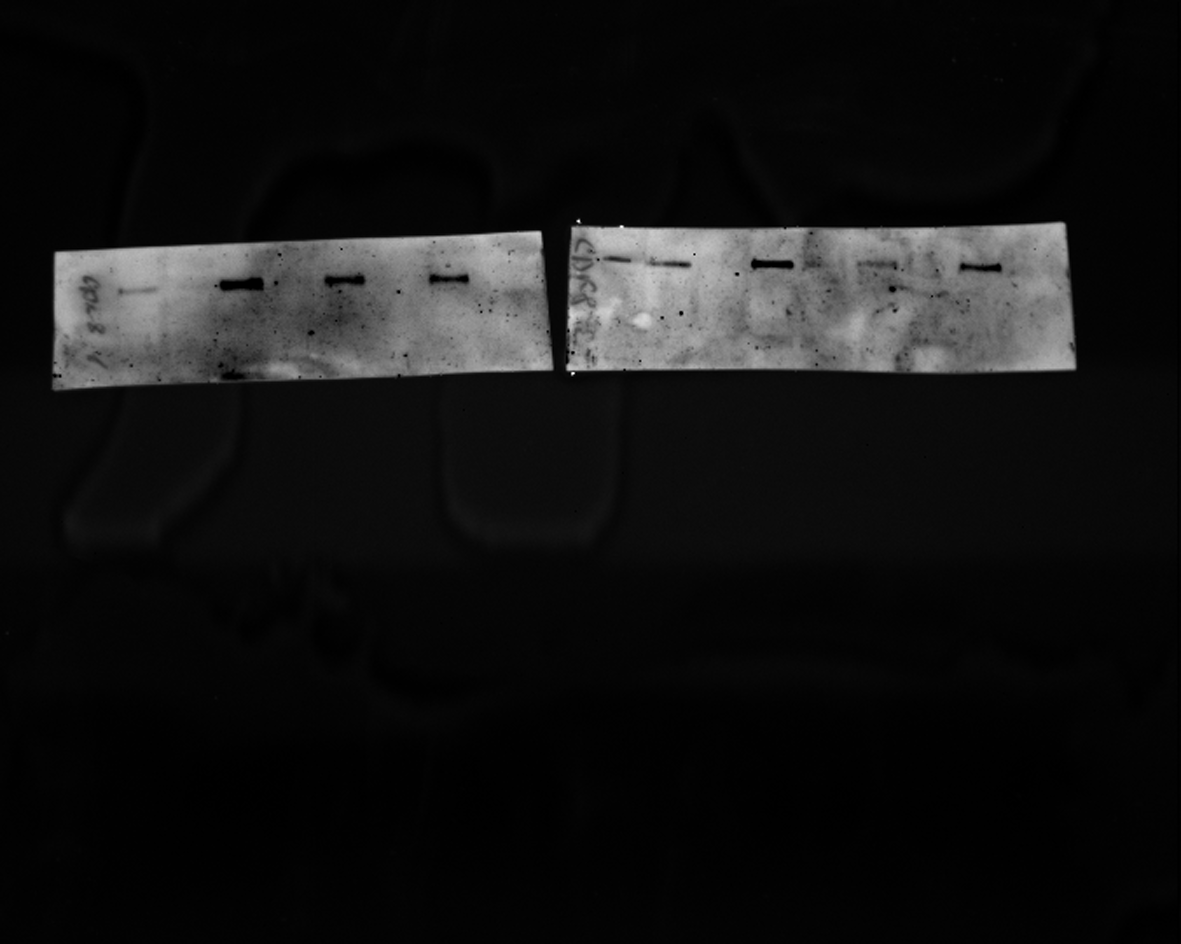

Supplement: Figure 2—figure supplement 1—source data 2. [file elife-96465-fig2-figsupp1-data2.zip › Figure 2-figure supplement 1-source data 2. Original files for western blot analysis displayed in Sup.Fig 2B/CDK8 (high exposure).tif]

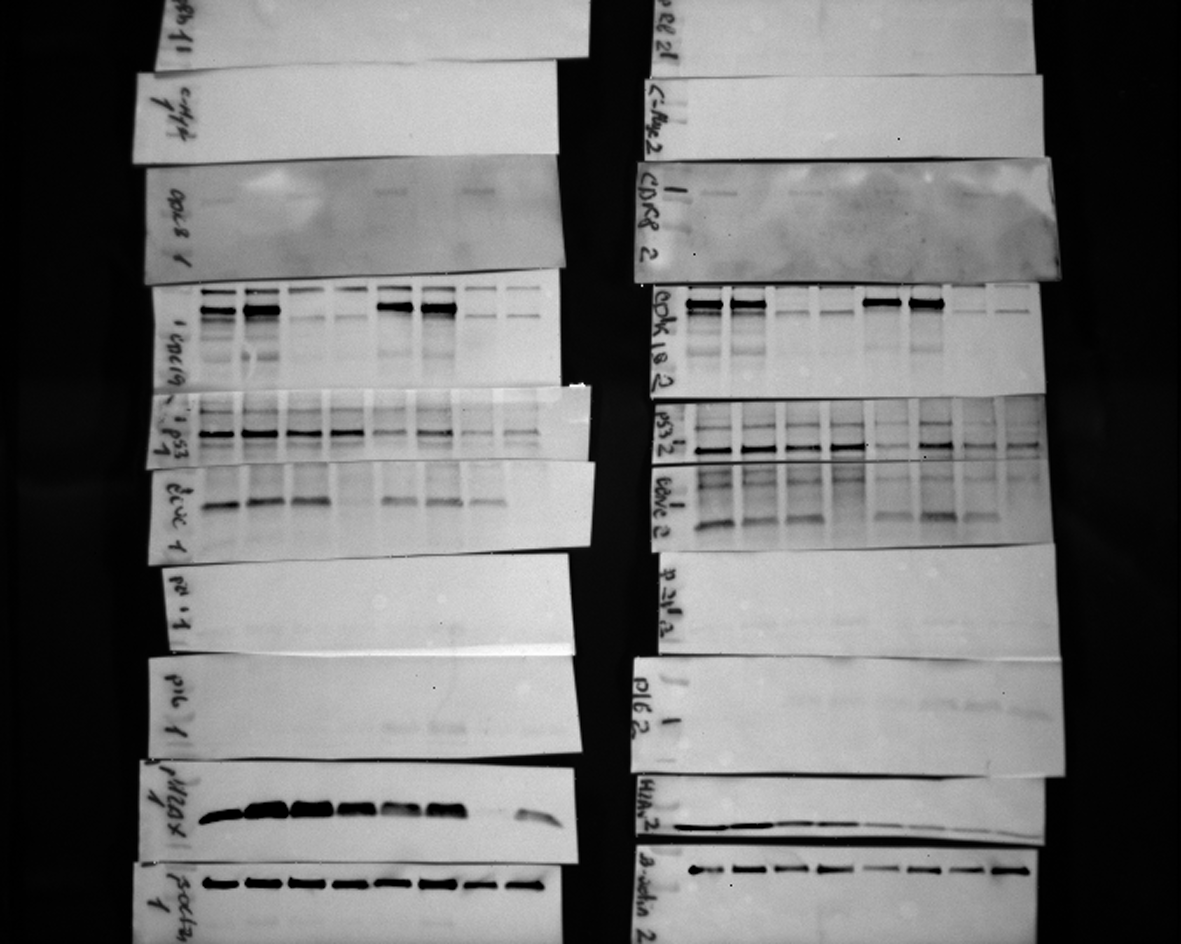

Supplement: Figure 2—figure supplement 1—source data 2. [file elife-96465-fig2-figsupp1-data2.zip › Figure 2-figure supplement 1-source data 2. Original files for western blot analysis displayed in Sup.Fig 2B/CDK8 + CDK19 + b-actin.tif]

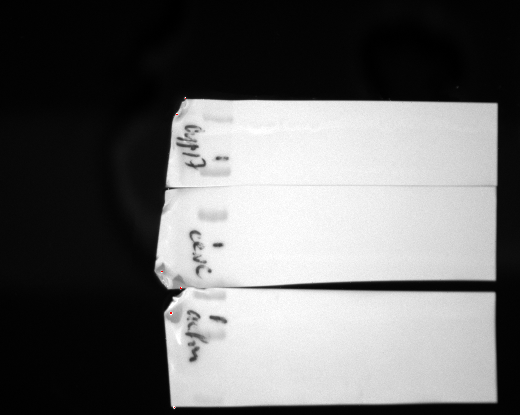

Supplement: Figure 6—source data 2. [file elife-96465-fig6-data2.zip › Figure 6 - source data 2. Original files for western blot analysis displayed in Figure 6B/CYP17A1 + b-actin (Membrane).tif]

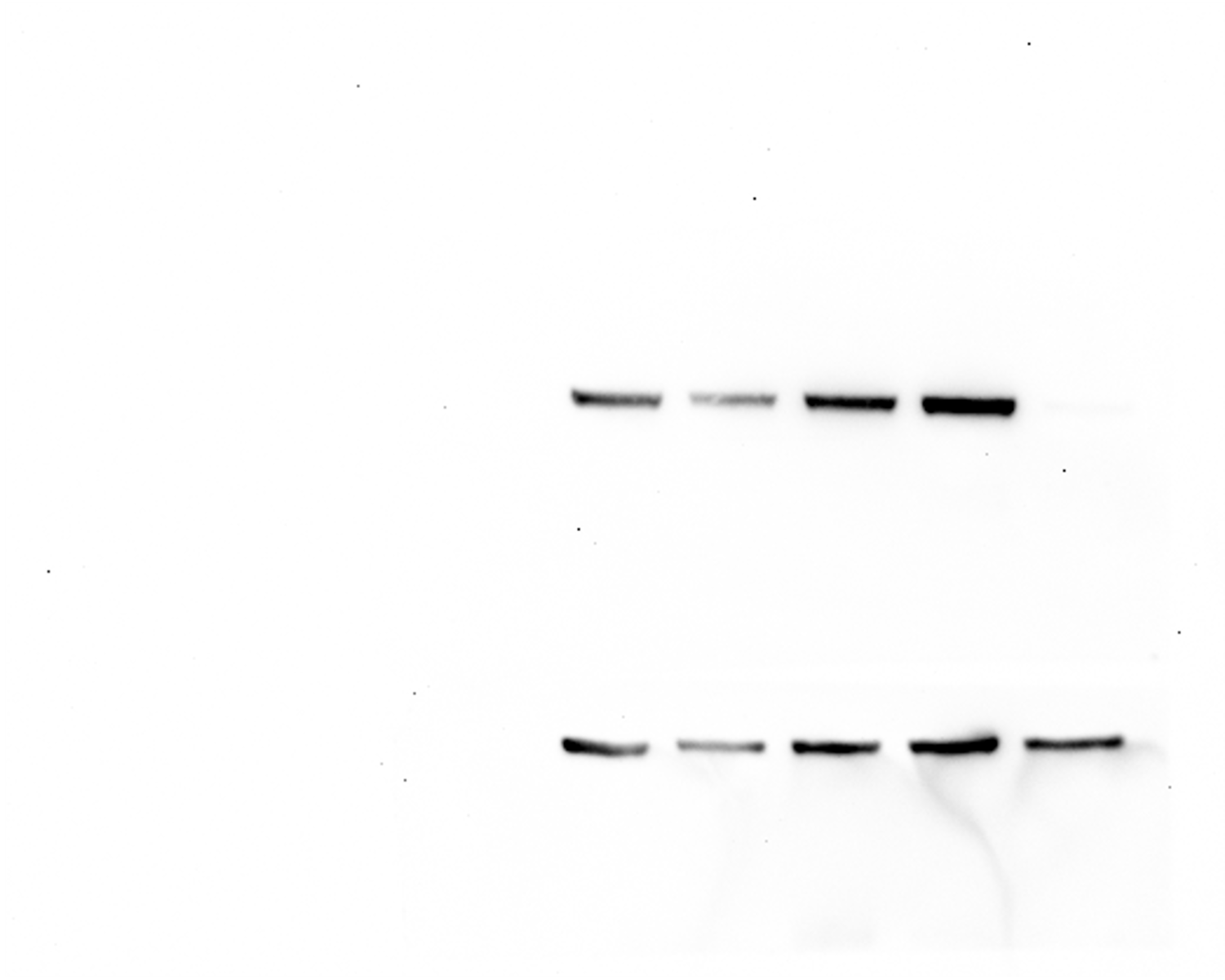

Supplement: Figure 6—source data 2. [file elife-96465-fig6-data2.zip › Figure 6 - source data 2. Original files for western blot analysis displayed in Figure 6B/CYP17A1 + b-actin.tif]

**CYP17A1**

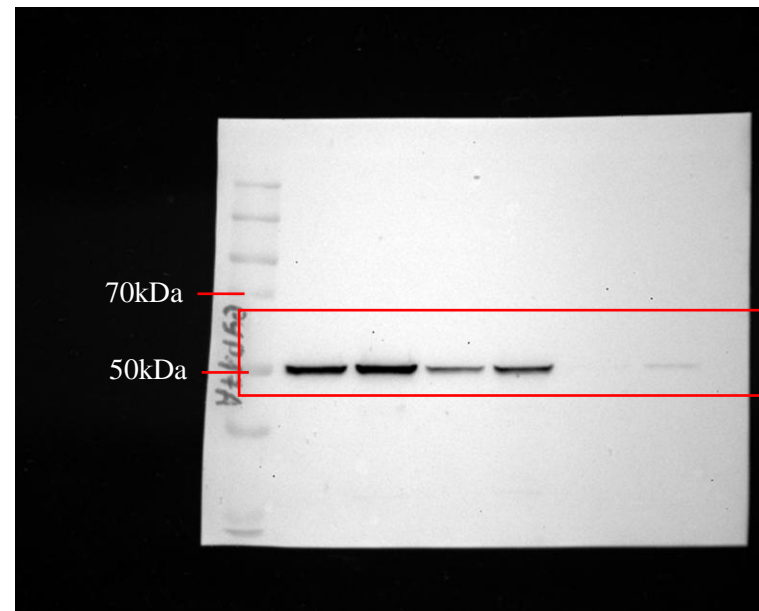

**b-actin**

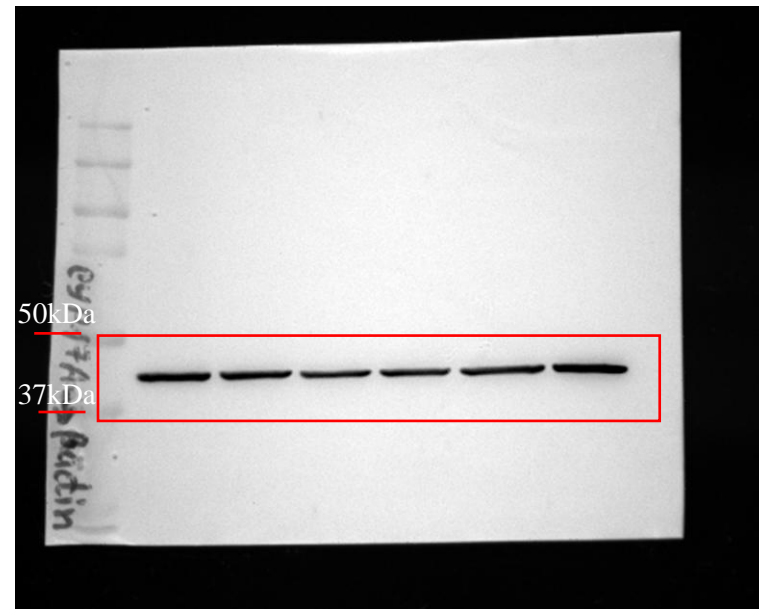

**Figure 7 - source data 1.** Original membranes corresponding to Figure 7, panel E.

Supplement: Figure 7—source data 1. [file elife-96465-fig7-data1.zip › Figure 7 - source data 1. PDF file containing original western blots for Figure 7E, indicating the relevant bands and treatments/Figure 7 - source data 1.pdf]

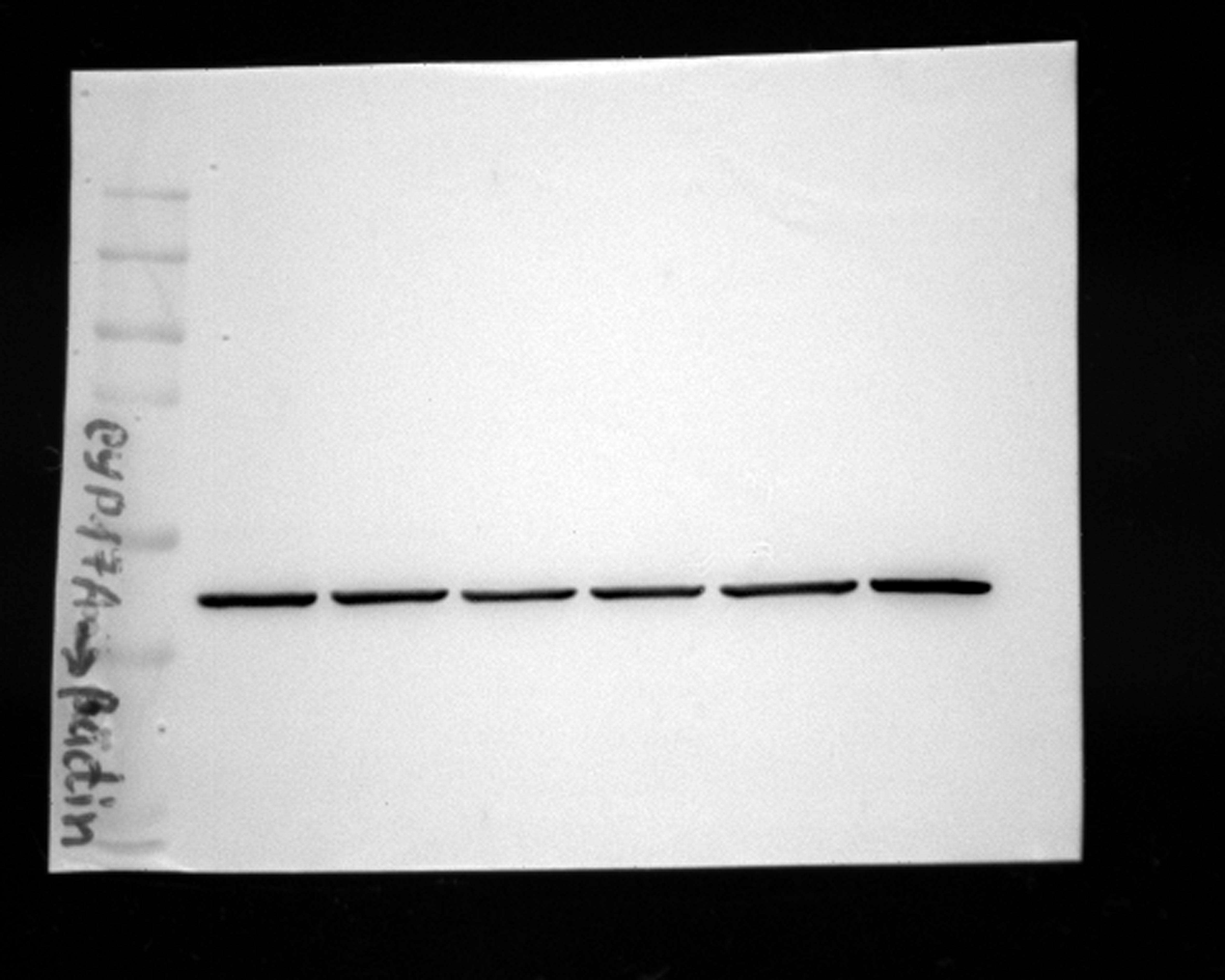

Supplement: Figure 7—source data 2. [file elife-96465-fig7-data2.zip › Figure 7 - source data 2. Original files for western blot analysis displayed in Figure 7E/b-actin.tif]

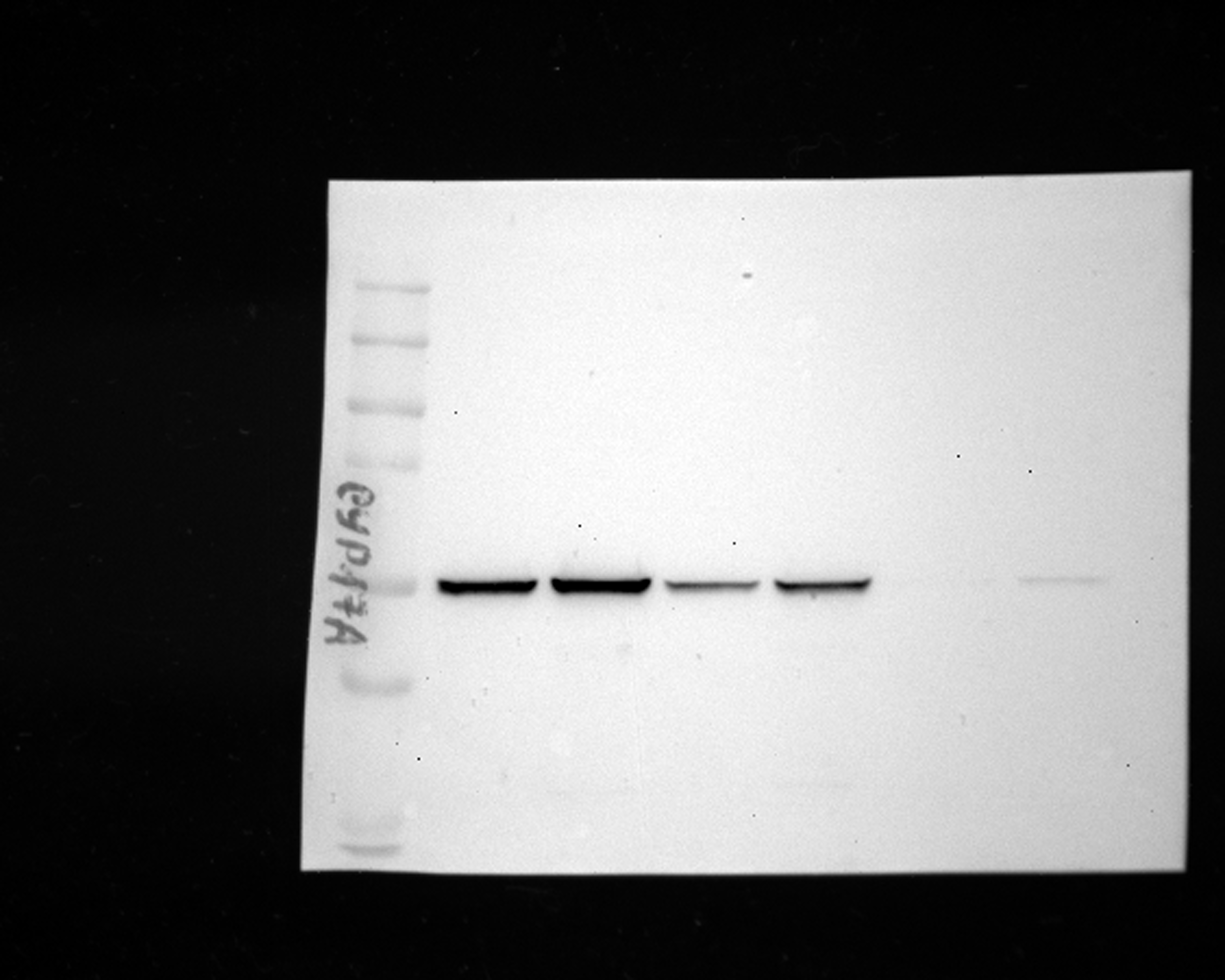

Supplement: Figure 7—source data 2. [file elife-96465-fig7-data2.zip › Figure 7 - source data 2. Original files for western blot analysis displayed in Figure 7E/CYP17A1.tif]
