# Supplementary material for: Knockout of cyclin-dependent kinases 8 and 19 leads to depletion of cyclin C and suppresses spermatogenesis and male fertility in mice
Source: eLife. 2025 Apr 2;13:RP96465. doi: 10.7554/eLife.96465 (PMC11964450; doi:10.7554/eLife.96465)
Supplement: Figure 1—figure supplement 1—source data 1. [file elife-96465-fig1-figsupp1-data1.zip › Figure 1-figure supplement 1-source data 1. PDF file containing original gel for Sup.Fig 1B, indicating the relevant bands and treatments/Fig1_suppl1B.pdf]

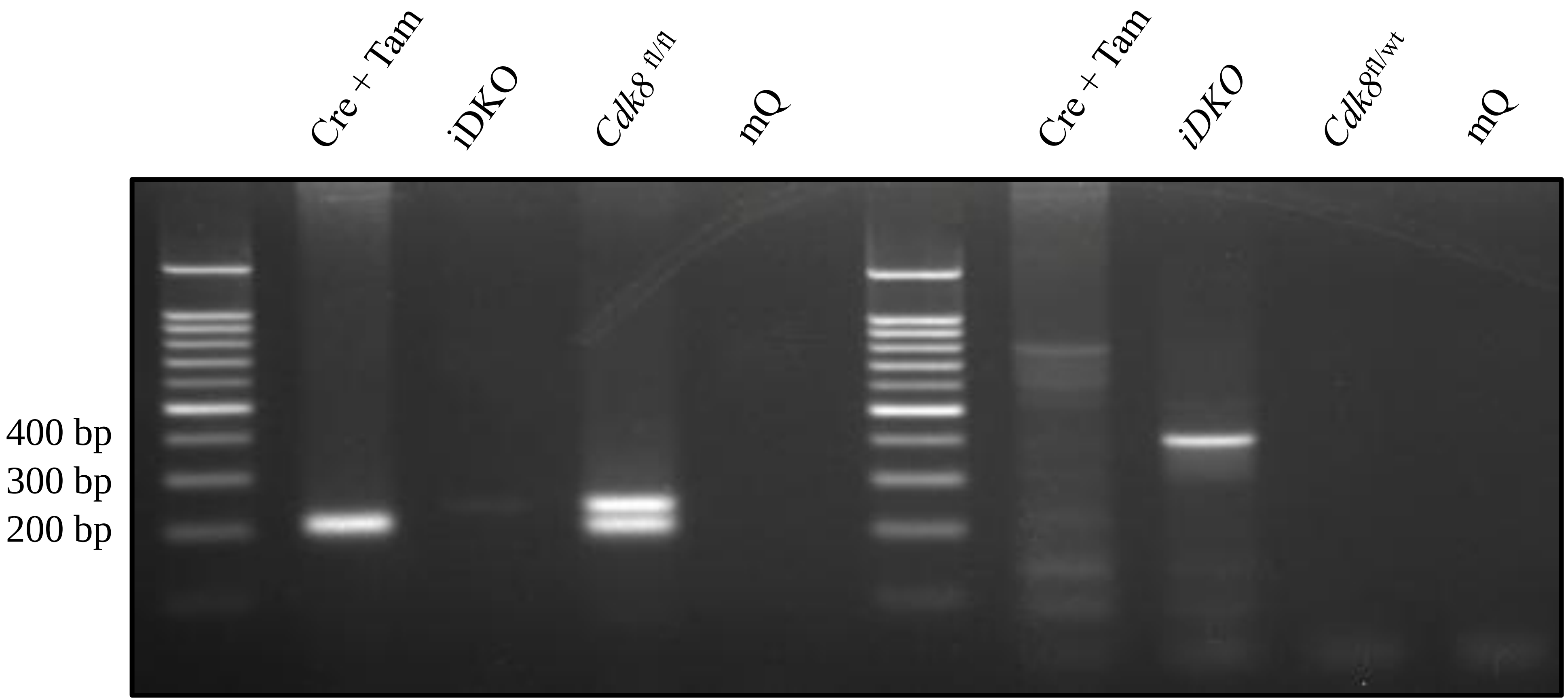

Figure 1-figure supplement 2 -source data 1. Original gel after electrophoresis corresponding to Figure 1, supplemental file 2.
