# Supplementary material for: Knockout of cyclin-dependent kinases 8 and 19 leads to depletion of cyclin C and suppresses spermatogenesis and male fertility in mice
Source: eLife. 2025 Apr 2;13:RP96465. doi: 10.7554/eLife.96465 (PMC11964450; doi:10.7554/eLife.96465)
Supplement: Figure 2—source data 1. [file elife-96465-fig2-data1.zip › Figure 2 - source data 1. PDF file containing original western blots for Figure 2D, indicating the relevant bands and treatments/Figure 2 - source data 1.pdf]

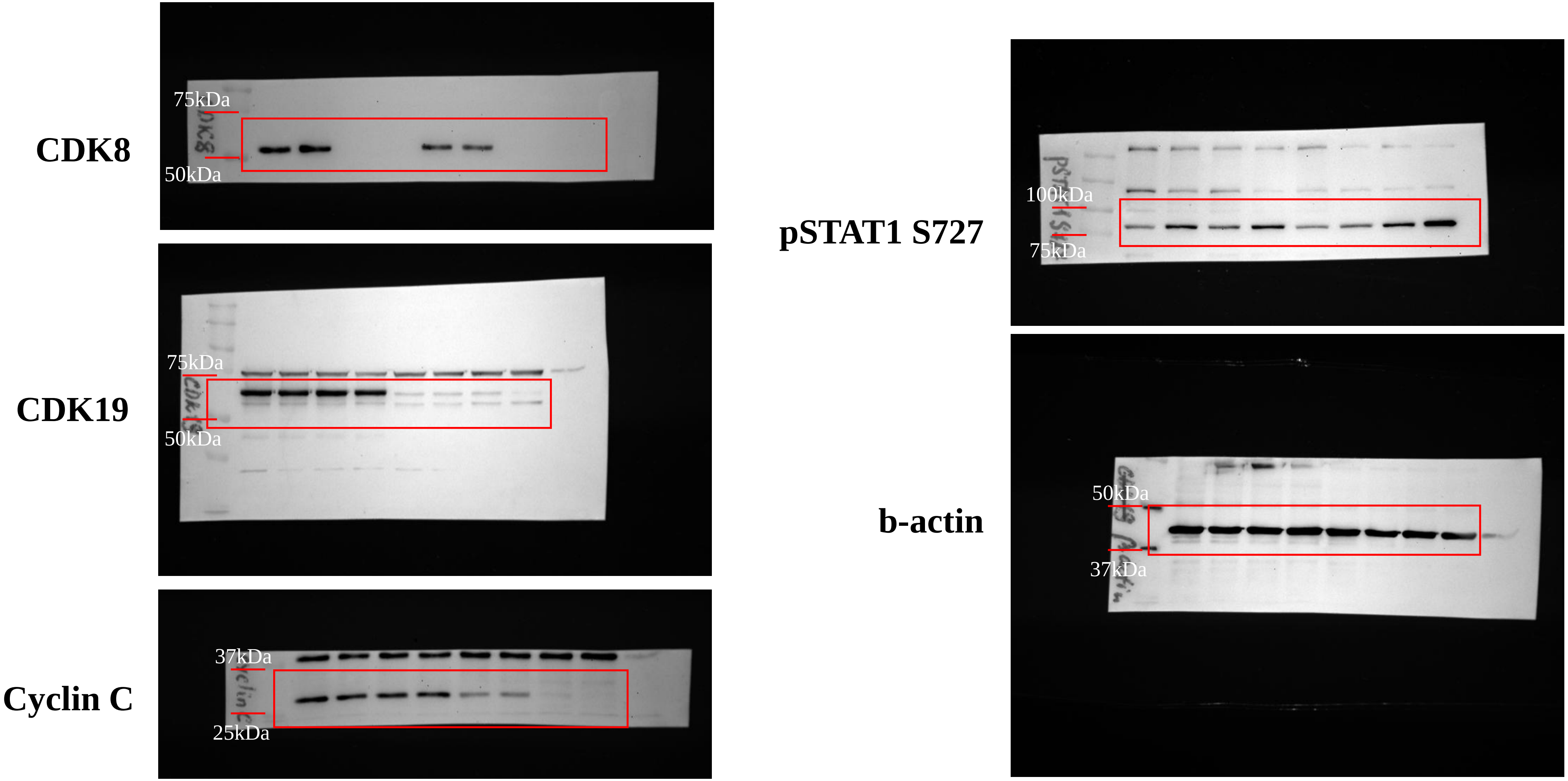

Figure 2 - source data 1. Original membranes corresponding to Figure 2, panel D.
