# Supplementary material for: Knockout of cyclin-dependent kinases 8 and 19 leads to depletion of cyclin C and suppresses spermatogenesis and male fertility in mice
Source: eLife. 2025 Apr 2;13:RP96465. doi: 10.7554/eLife.96465 (PMC11964450; doi:10.7554/eLife.96465)
Supplement: Figure 2—figure supplement 1—source data 1. [file elife-96465-fig2-figsupp1-data1.zip › Figure 2-figure supplement 1-source data 1. PDF file containing original western blots for Sup.Fig 2B, indicating the relevant bands and treatments/Fig2_suppl2B.pdf]

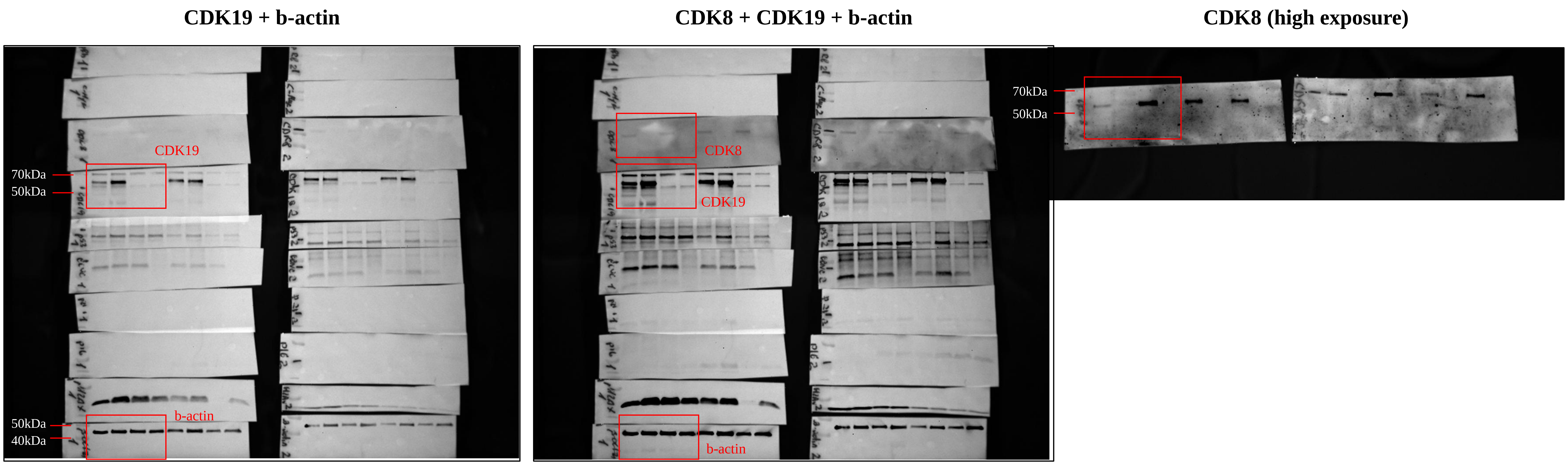

Figure 1-figure supplement 7-source data 1. Original membranes corresponding to Figure 1, supplemental file 7.
