# Supplementary material for: Knockout of cyclin-dependent kinases 8 and 19 leads to depletion of cyclin C and suppresses spermatogenesis and male fertility in mice
Source: eLife. 2025 Apr 2;13:RP96465. doi: 10.7554/eLife.96465 (PMC11964450; doi:10.7554/eLife.96465)
Supplement: Supplementary file 4. [file elife-96465-supp4.pdf]

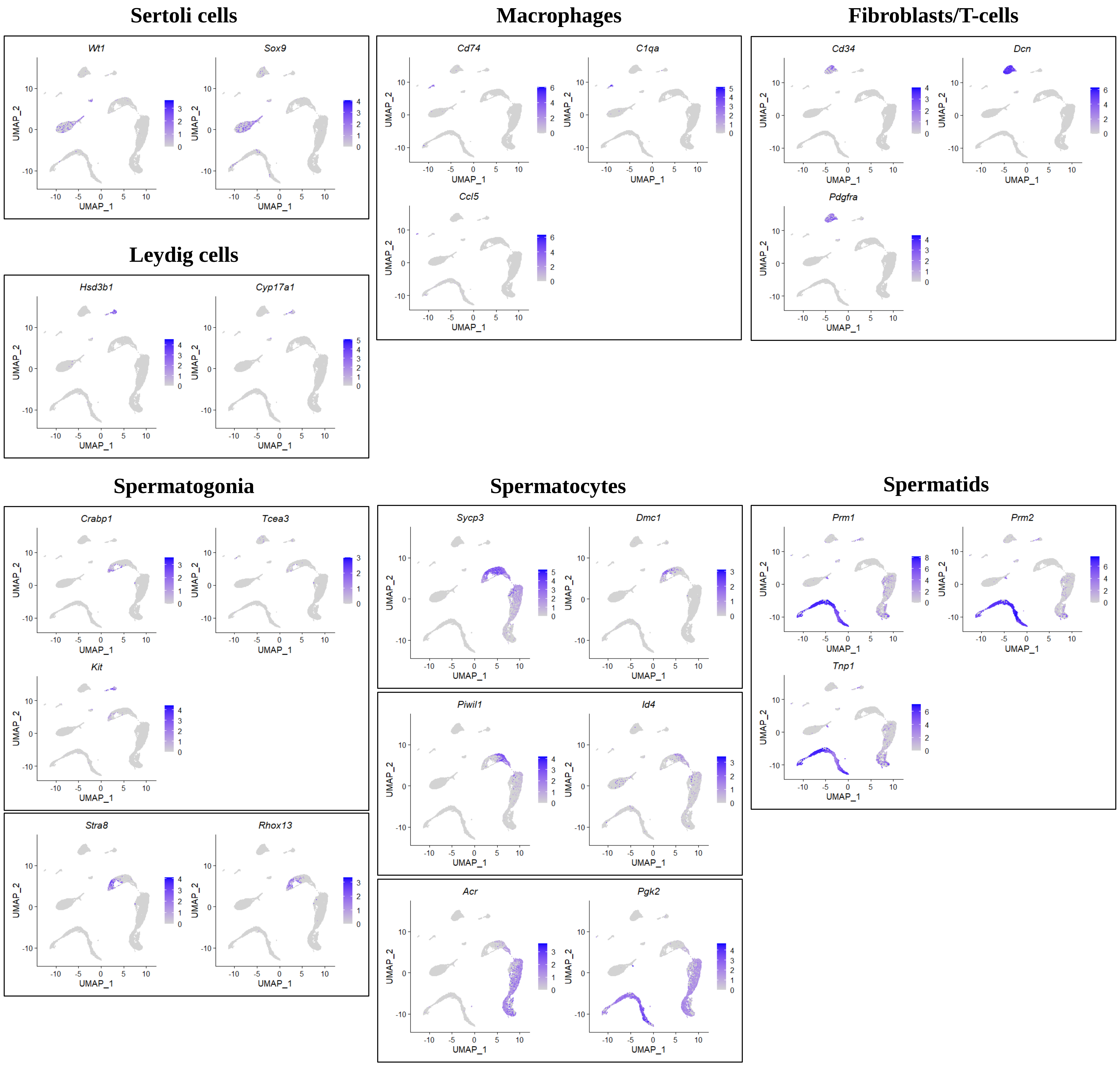

Clusterization markers for main cell types
